# Development and characterization of phasor-based analysis for FLIM to evaluate the metabolic and epigenetic impact of HER2 inhibition on squamous cell carcinoma cultures

**DOI:** 10.1117/1.JBO.26.10.106501

**Published:** 2021-10-09

**Authors:** Dan L. Pham, Christina R. Miller, Molly S. Myers, Dominick M. Myers, Laura A. Hansen, Michael G. Nichols

**Affiliations:** a Creighton University, Department of Physics, Omaha, Nebraska, United States; b Creighton University, Department of Biomedical Sciences, Omaha, Nebraska, United States

**Keywords:** squamous cell carcinoma, phasor fluorescence lifetime imaging microscopy, human epidermal growth factor receptor 2, AG825, metabolic imaging, two-photon microscopy, NADH, NAD(P)H

## Abstract

**Significance**: Deranged metabolism and dysregulated growth factor signaling are closely associated with abnormal levels of proliferation, a recognized hallmark in tumorigenesis. Fluorescence lifetime imaging microscopy (FLIM) of endogenous nicotinamide adenine dinucleotide (NADH), a key metabolic coenzyme, offers a non-invasive, diagnostic indicator of disease progression, and treatment response. The model-independent phasor analysis approach leverages FLIM to rapidly evaluate cancer metabolism in response to targeted therapy.

**Aim**: We combined lifetime and phasor FLIM analysis to evaluate the influence of human epidermal growth factor receptor 2 (HER2) inhibition, a prevalent cancer biomarker, on both nuclear and cytoplasmic NAD(P)H of two squamous cell carcinoma (SCC) cultures. While better established, the standard lifetime analysis approach is relatively slow and potentially subject to intrinsic fitting errors and model assumptions. Phasor FLIM analysis offers a rapid, model-independent alternative, but the sensitivity of the bound NAD(P)H fraction to growth factor signaling must also be firmly established.

**Approach:** Two SCC cultures with low- and high-HER2 expression, were imaged using multiphoton-excited NAD(P)H FLIM, with and without treatment of the HER2 inhibitor AG825. Cells were challenged with mitochondrial inhibition and uncoupling to investigate AG825’s impact on the overall metabolic capacity. Phasor FLIM and lifetime fitting analyses were compared within nuclear and cytoplasmic compartments to investigate epigenetic and metabolic impacts of HER2 inhibition.

**Results**: NAD(P)H fluorescence lifetime and bound fraction consistently decreased following HER2 inhibition in both cell lines. High-HER2 SCC74B cells displayed a more significant response than low-HER2 SCC74A in both techniques. HER2 inhibition induced greater changes in nuclear than cytoplasmic compartments, leading to an increase in NAD(P)H intensity and concentration.

**Conclusions:** The use of both, complementary FLIM analysis techniques together with quantitative fluorescence intensity revealed consistent, quantitative changes in NAD(P)H metabolism associated with inhibition of growth factor signaling in SCC cell lines. HER2 inhibition promoted increased reliance on oxidative phosphorylation in both cell lines.

## 1 Introduction

Cellular proliferation is strictly regulated by both the intracellular and extracellular environment. Although cellular metabolism is responsible for energy generation and macromolecule synthesis in cell growth and division, the growth-factor signaling pathway drives the cell cycle and induces epigenetic modifications that initiate and facilitate proliferation. Thus deranged metabolism and dysregulated growth factor signaling are closely associated with abnormal levels of proliferation, a commonly recognized hallmark in tumorigenesis. Within the growth factor receptor family, human epidermal growth factor receptor 2 (HER2) is overexpressed in breast, skin, gastric, and gastroesophageal cancers, with high-HER2 activity associated with worse prognosis and treatment outcome.^1^ HER2 targeting is a well-accepted treatment regimen for HER2 positive breast cancer, and HER1/EGFR targeting is commonly used in head and neck squamous cell carcinoma (HNSCC), where overexpression of EGFR occurs in 80% to 90% of tumors and is similarly associated with poor outcomes.^2^ The use of HER2 therapy in cutaneous squamous cell carcinoma (cSCC) has not been thoroughly explored, however, due to skin cancer’s commonly benign nature, most do not require adjuvant treatment. However, skin cancer accounts for more new cases diagnosed than every other cancer combined and SCCs are the second most common subtype.^3^^–^^5^ The majority of skin cancer cases can be attributed to ultraviolet (UV) radiation, and previous studies have found that chronic UV exposure stimulates HER2 dimerization and activation through UV-induced reactive oxygen species (ROS).^6^^–^^9^

Cancer cells, following the Warburg effect, often display significantly higher rates of glycolysis and glucose consumption compared to normal cells, providing the advantages of fast energy production while sparing materials for macromolecular synthesis.^10^ These changes in metabolism, in turn, affect the concentration and binding activities of metabolic coenzymes such as nicotinamide adenine dinucleotide (NADH) and flavin adenine dinucleotide (FAD). During cellular metabolism, NADH and ${\mathrm{FADH}}_{2}$ are created during glycolysis and the Krebs cycle, and oxidized to NAD+ and FAD through oxidative phosphorylation. NADH and FAD are endogenous fluorophores, and their potential for diagnostic imaging has been a dynamic field of study since the original pioneering work of Chance et al.^11^^,12^ Because the optical properties of NADH and NADPH are identical, the blue autofluorescent signal in metabolic imaging is commonly referred to as NAD(P)H.

Previous research has showed that the optical redox ratio, the ratio of fluorescence intensity of NAD(P)H to that of FAD, is sensitive to changes in cellular metabolism induced during malignant transformation, metastatic progression, and is significantly affected by expression levels of estrogen receptor (ER) and HER2.^13^^–^^15^ Treatment with trastuzumab, a specific inhibitor of HER2, was found to significantly reduce the optical redox ratio, demonstrating that optical monitoring may be used to measure changes in metabolism induced by the activity of HER2.^16^^,^^17^ In our study, we used AG825, a competitive inhibitor of tyrosine kinase, as a HER2-specific inhibitor.

Because the FAD fluorescence signal is weaker than NAD(P)H in tissue and two channel metabolic imaging significantly increases time and cost,^18^^,^^19^ in this study, we used fluorescence lifetime imaging microscopy (FLIM) of NAD(P)H alone to specifically investigate the impact of the HER2 on two human HNSCC cultures, SCC74A and SCC74B, expressing low and high HER2, respectively.^20^ FLIM allows for quantification of not only the fluorescence intensity but also the excited-state lifetime, which is sensitive to the local environment and biological role of NAD(P)H. Free NADH self-quenches and has a short lifetime of about 0.4 ns. Meanwhile, protein-bound NADH in its extended form has a longer lifetime of ∼3.4  ns. Shifts in the relative concentrations of free and bound NADH, which occur with changes in the balance between NADH production by glycolysis and the Krebs cycle, and its oxidation by the electron transport chain, result in measurable changes in both the intensity and the mean lifetime of NAD(P)H. These changes are also clearly observable using the phasor FLIM approach, which has the benefit of being model independent and permits rapid data analysis.^21^^,^^22^

In this study, we combined two complementary approaches to quantitatively analyze FLIM images: fluorescence decay fitting to obtain the mean fluorescent lifetime and phasor-based analysis of the bound NAD(P)H fraction. Quantitative analysis of the fluorescence intensity was also used to infer relative changes in NAD(P)H concentration. We determined the relationship between the bound NAD(P)H fraction and mean lifetime measurements and quantified the impact of AG825 on SCC metabolism. Using this combined analysis approach, we considered whether metabolic imaging of NAD(P)H could reveal the dynamic relationship between HER2 inhibition and altered metabolism in squamous cell carcinoma (SCC). To characterize the metabolic dynamic range of each cell line, we assessed the variation due to mitochondrial inhibitor (rotenone) and uncoupler (carbonyl cyanide p-trifluoro-methoxyphenyl hydrazone or FCCP) treatments with respect to routine culture (RC) conditions with and without HER2 inhibition. Given the interplay between the various cellular roles of NAD(P)H, including its role in metabolism and gene expression regulation, we characterized NAD(P)H changes in cytoplasmic and nuclear compartments independently.^23^^,^^24^ Overall, we demonstrate that the combination of the mean fluorescence lifetime, intensity and the bound NAD(P)H fraction provides a quantitative approach to non-invasively measure, interpret, and compare the impact of growth factor expression within SCC *in vitro.*

## 2 Materials and Methods

### 2.1 Cell Culture

The UM-SCC74A cell line (low HER2, derived from a tongue tumor) and UM-SCC74B (high HER2, from a 1-year recurrence) were cultured as described previously.^17^^,^^25^ Briefly, cells were cultured in DMEM, supplemented with 1% penicillin–streptomycin and 10% fetal bovine serum (Life Technologies, Carlsbad, CA) and maintained at 37°C and 5% ${\mathrm{CO}}_{2}$ in a humidified incubator.

### 2.2 Metabolic Treatments and HER2 Inhibition

SCC74A and SCC74B cells were plated on coverslips 3 days before experiment to ensure 80% confluency for imaging. Prior to imaging, cells were washed and placed in modified Tyrodes imaging buffer (135 mM NaCl, 5 mM KCl, 1 mM ${\mathrm{MgCl}}_{2}{\cdot 6H}_{2}O$, 1.8 mM ${\mathrm{CaCl}}_{2}{\cdot 2H}_{2}0$, 20 mM HEPES, 5 mM glucose, and 0.25% bovine serum albumin). To assess metabolic changes in response to HER2 inhibition, cells were treated with 30  μM AG825 or DMSO (0.1% vehicle control), in culture media at 37°C for 20 h. Following treatment, coverslips were washed and placed in imaging chamber with modified Tyrodes imaging buffer, with or without metabolic treatments. To assess metabolic function, cells were treated with 1  μM rotenone (mitochondrial inhibitor) or 10  μM FCCP (mitochondrial uncoupler) in imaging buffer for 15 min at 37°C and 5% ${\mathrm{CO}}_{2}$. RC cells were imaged in imaging buffer alone. All reagents were from Sigma-Aldrich (St. Louis, MO), unless otherwise indicated.

### 2.3 NAD(P)H Fluorescence Lifetime Imaging

**Fig. 1:**
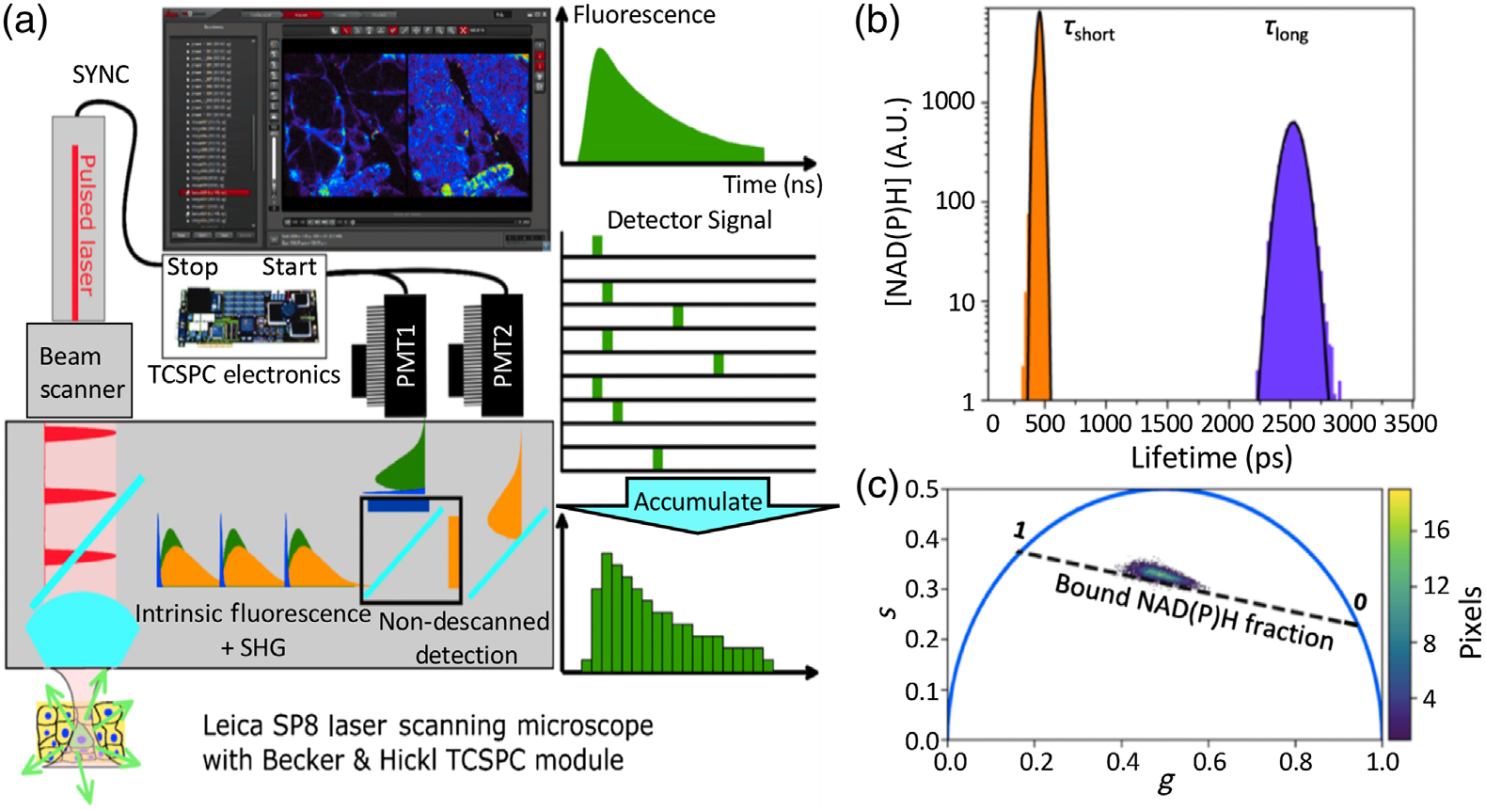
System setup for metabolic FLIM imaging with two analysis approaches. (a) Time-correlated single-photon counting FLIM is used to measure fluorescence decays F(x,y,t) and produce lifetime (τ) images. (b) Fluorescence decays for each pixel are fit with single- and double-exponential decay curves and concentration histograms typically identify two significant pools associated with short- and long-lifetime NAD(P)H, respectively. (c) Alternatively, for phasor-FLIM analysis, sine and cosine transforms of the decay curve are computed using the fundamental frequency of the pulsed laser system of 100 MHz. The bound NAD(P)H fraction is measured along the dashed line ranging from 0 (free) to 1 (enzyme bound) NAD(P)H, as determined from solution measurements. Image processing optionally segments cytoplasmic and nuclear regions, otherwise, whole cell averages are calculated.

Fluorescence intensity and lifetime imaging of two-photon-excited NAD(P)H and flavin mononucleotide were performed using the 740-nm mode-locked femtosecond pulse train of a Spectra Physics MaiTai Ti:S laser on a Leica TCS SP8 MP multiphoton laser scanning confocal microscope (Leica Microsystems, Germany) equipped with a Leica HC PL APO CS2 40X/1.3 NA oil immersion objective [Fig. 1(a)]. Blue and yellow endogenous non-descanned fluorescence was separated using a 500-nm long pass dichroic mirror and isolated with HQ 460/80-2p, and HQ 580/60-2p band-pass filters (Chroma Technology, Bellows Falls, VT, USA), respectively, then detected with high-sensitivity super HyD detectors and a time-correlated single-photon counting module (830 SPC, Becker and Hickl, Berlin, Germany). Coverslips were maintained at 37°C±1°C during imaging using a transparent stage heater (Cell MicroControls, Norfolk, VA). The imaging field of view was 138  μm×138  μm (128×128  pixels) with a line scan rate of 400 Hz (dwell time of 19.5  μs/pixel), a line average of 3 and a total acquisition time of 120 s. The laser power at the sample was kept the same across different treatment conditions in every replicate of experiment. The laser power was optimized for each replicate to ensure an appropriate photon counts for fluorescence lifetime fitting but was never higher than 1.6 mW to minimize photobleaching.

### 2.4 Fluorescence Lifetime and Intensity Analysis

Metabolic image analysis of NAD(P)H fluorescence lifetimes and intensity was performed following methods described previously.^26^ To better facilitate analysis of distinct subcellular regions (nucleus or cytoplasmic), a lifetime FLIM analysis program was developed in LabView^®^. Background regions were excluded from the analysis by thresholding. Regions of interest (ROIs) outlining nuclear and cytoplasmic regions were manually defined and analyzed. Fluorescence decay data for each pixel were binned (3×3) to ensure a minimum of 20,000 photons contributed to the decay fitting. Fits to single- and double-exponential decays were performed, and the goodness of fit was compared using an F-test as described previously to determine whether the extra fitting parameters used in the double exponential fit led to sufficient improvement in the fit to justify their inclusion.^27^ Lifetime fitting was done using a Levenberg–Marquardt algorithm written in C and implemented within Labview^®^. Lifetime histograms of all the measured fluorescence lifetimes within an ROI were compiled with 50 ps binning (approximately matching the time resolution of the detectors), and distinct lifetime populations were characterized by fitting to normal distributions to determine the mean and standard error of the mean for each lifetime pool [Fig. 1(b)]. In the case of a double exponential decay, the mean fluorescence lifetime was computed as

$$\tau_{m}=\frac{A_{1}\tau_{1}+A_{2}\tau_{2}}{A_{1}+A_{2}},$$

where A and τ are the amplitude and lifetime, respectively, associated with each decay. The changes in NAD(P)H fluorescence intensity were quantified by normalizing the average fluorescence intensity of treated cells to the average intensity of vehicle control cells (RC DMSO) measured on the same day. To correct for possible photobleaching over the course of image acquisition, the average fluorescence intensity of each frame was calculated for the entire acquisition period. The measured intensity was then normalized by the bleaching correction factor, defined as the ratio of the average fluorescence intensity to the intensity of the first frame. This correction was typically <10% and there was no significant variation in photobleaching under the various treatments. Finally, variation in the total NAD(P)H concentration was estimated from the ratio of the fluorescence intensity and mean fluorescence lifetime.

### 2.5 Phasor-FLIM Analysis

Model-independent phasor FLIM analysis was performed using custom analysis code written in the python programming language to facilitate analysis of arbitrary regions as with the lifetime analysis [Fig. 1(c)]. Briefly, 3×3  pixels were binned and the resulting fluorescence decay F(x,y,t) was transformed using sine and cosine transforms:

$$s(x,y,\omega )=\frac{\int F(x,y,t)\sin(\omega t)dt}{\int F(x,y,t)dt}\;g(x,y,\omega )=\frac{\int F(x,y,t)\cos(\omega t)dt}{\int F(x,y,t)dt},$$

where ω=2π(100  MHz)^21^^,^^22^ and the numerical integral was taken over one complete period T=2π/ω=10  ns. The transformation was corrected for the instrument response function estimated from the fluorescence decay of the brightest pixel in the image, using the method described by Štefl et al.^28^ A two-dimensional phasor histogram was then calculated.

When there are several fluorescent species with distinct lifetimes, the compounded fluorescence decay may be approximated by a sum of exponentials. Hence, the phasor coordinates will depend on the product of the fluorescence lifetime and amplitude of each species. In the general case of a sum of n exponential decays, the coordinates are^29^^–^^32^

$$s(\omega )=\frac{1}{N}\overset{n}{\underset{i=1}{\sum }}A_{i}\tau_{i}(\frac{\omega \tau_{i}}{1+{\left(\omega \tau_{i}\right)}^{2}}),$$

and

$$g(\omega )=\frac{1}{N}\overset{n}{\underset{i=1}{\sum }}A_{i}\tau_{i}(\frac{1}{1+{\left(\omega \tau_{i}\right)}^{2}}),$$

where $N=\overset{n}{\underset{i=1}{\sum }}A_{i}\tau_{i}$.

Previous papers have determined the fluorescence lifetimes of free and bound NADH using NADH binding enzyme LDH to be $\tau_{1}=0.4\;\mathrm{ns}$ and $\tau_{2}=3.4\;\mathrm{ns}$, respectively.^33^^,^^31^ Translating these characteristic lifetimes to phasor coordinates results in a metabolic trajectory extending from $p_{1}$ (s=0.234, g=0.906) −100% free NADH to $p_{2}$ (s=0.381, g=0.130) −100% bound NADH when the phasor transform was performed at 100 MHz. We used 100 MHz as our standard transform frequency for compatibility with another laser used for *in vivo* imaging in our lab as the phasor endpoints vary with the frequency of the transform.^34^ The bound NAD(P)H phasor endpoint was also consistent with that of flavin mononucleotide, which enabled an easier, routine calibration.

We then assigned the global free-bound-NAD(P)H phasor axis as the line connecting $p_{1}$ and $p_{2}$ [Fig. 1(c)]. For each pixel in the image with phasor coordinate $\left(g_{i},s_{i}\right)$, we found its projection onto the global phasor axis $\left(g_{i^{\prime }},s_{i^{\prime }}\right)$ and calculated the distance $l_{i}$ between the projected coordinates and $p_{1}$. The distance $l_{i}$ was then normalized to the absolute length of the phasor axis and determined as the bound NAD(P)H fraction, ranging from 0 [free NAD(P)H] to 1 [bound NAD(P)H]. Since the free and bound states are both reasonably approximated by single exponential decays with the aforementioned lifetimes, it can readily be shown that the length of the entire phasor axis is given by

$$L={\left(\frac{1}{1+{\left(\omega \tau_{1}\right)}^{2}}+\frac{1}{1+{\left(\omega \tau_{2}\right)}^{2}}-2\frac{1+(\omega \tau_{1})(\omega \tau_{2})}{\left(1+{\left(\omega \tau_{1}\right)}^{2})(1+{\left(\omega \tau_{2}\right)}^{2}\right)}\right)}^{1/2},$$

and the normalized length along the phasor axis is given by

$$\frac{l_{i}}{L}=\frac{A_{2}\tau_{2}}{A_{1}\tau_{1}+A_{2}\tau_{2}}.$$

For each field of view, a histogram of the bound NAD(P)H fraction was fit to a sum of Gaussians (either one or two, if statistically justified by an F-test) to determine the mean bound NAD(P)H fraction for the image. The average bound NAD(P)H fraction was computed and compared across all treatment conditions.

The bound NAD(P)H fraction determined in this way represents the fraction of the fluorescence signal that is associated with the bound state. Given the longer lifetime and greater fluorescence quantum yield, an enzyme-bound NAD(P)H molecule will contribute a greater fraction of the overall signal than a free NAD(P)H molecule, just as enzyme-bound NAD(P)H contributes more significantly to the mean fluorescence lifetime. However, while the mean fluorescence lifetime and the phasor bound fraction are both candidate reporters for monitoring cellular metabolism, they are clearly not equivalent—even in the case when fluorescence decay is well described by a sum of exponentials.

### 2.6 Image Segmentation to Assess Nuclear and Cytoplasmic Compartments

**Fig. 2:**
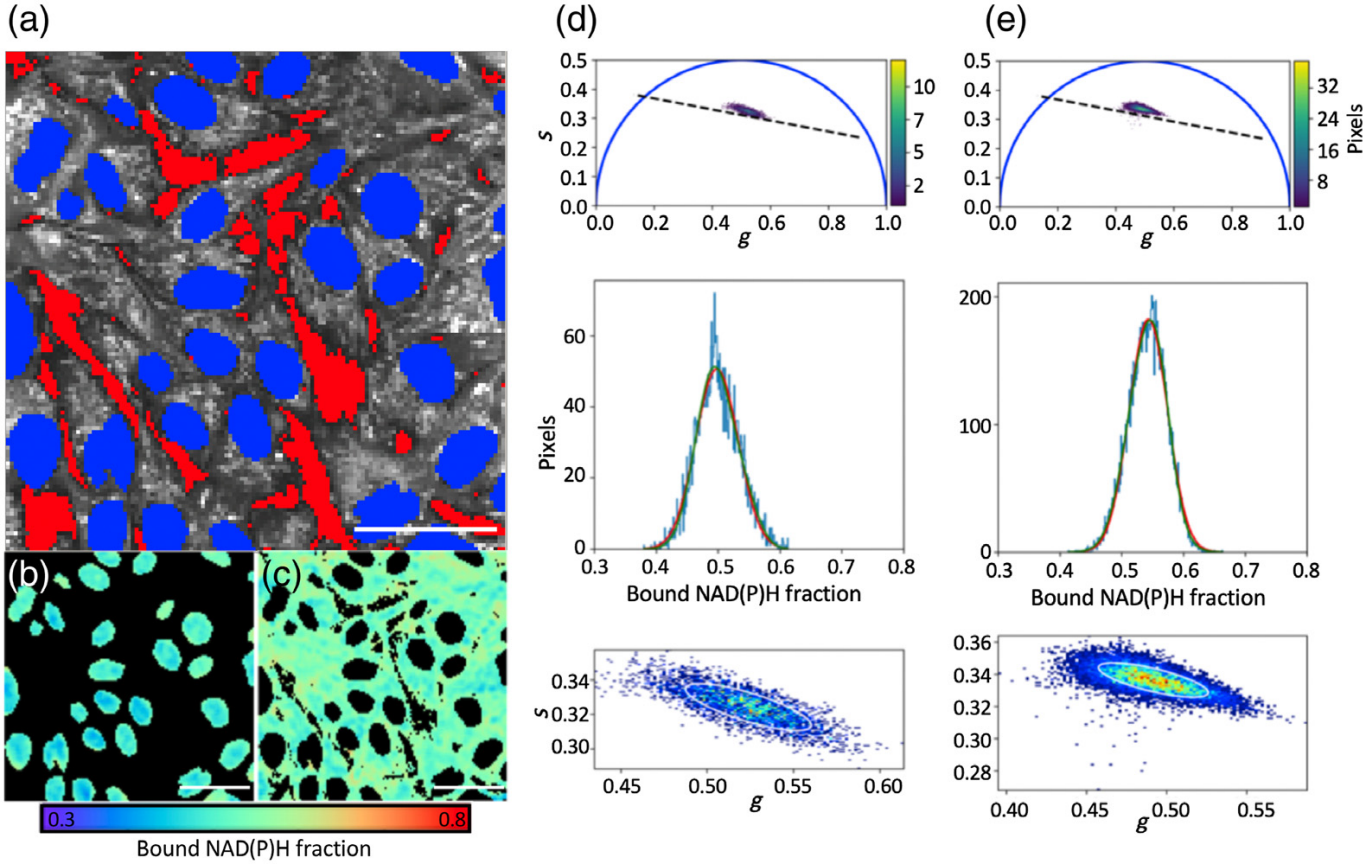
Phasor analysis of cytoplasmic and nuclear bound NAD(P)H fraction. (a) Image segmentation using a custom Python script allows separate phasor analyses of nuclear and cytoplasmic compartments. Intensity-based threshold was first applied onto the image to eliminate background (red), then nuclear ROIs were manually identified (blue). Cytoplasmic region was defined as secondary object in gray (scaled by intensity). (b), (c) Bound NAD(P)H fraction images of nuclear and cytoplasmic regions, respectively. The bound NAD(P)H fraction was calculated for each pixel based on the phasor analysis approach described above. (d), (e) 2D phasor histograms and bound NAD(P)H histogram distributions for nuclear and cytoplasmic regions, respectively. Nuclear region has lower bound NAD(P)H fraction compared to cytoplasmic region. Scale bar is 50  μm.

To independently assess changes in the bound NAD(P)H fraction within the nuclear and cytoplasmic compartments, a Python script was written to produce nuclear and cytoplasmic masks based on free-hand ROI drawing (Fig. 2). Nuclear ROIs were drawn based on the reduced endogenous NAD(P)H fluorescence intensity and labeled in blue [Fig. 2(a)]. The background region (red) was excluded based on a user-defined threshold. Pixels lying outside both the nuclear region and the background regions were assigned to the cytoplasmic image mask. Phasor FLIM analysis was applied and the 2D phasor distribution was determined for both regions [Figs. 2(b)–2(e)]. Additionally, the bound NAD(P)H fraction was computed for each pixel in the image, and the mean bound NAD(P)H fraction was determined for each compartment within the image.

### 2.7 Statistical Analysis

The R programming language was used for statistical analysis.^35^ There are several main factors in our experimental design: cell lines (with two levels SCC74A and SCC74B), HER2 inhibition treatment (with two levels RC and AG825), and metabolic treatments (with three levels DMSO, FCCP, and rotenone). Since interactions are possible among these factors, the data are first fit to a linear model (lm class) to determine the significance of the interaction terms. When the interaction terms were not significant, we performed a type II factorial ANOVA. Otherwise, we used a type III factorial ANOVA. In either case, the factorial test was followed by Tukey-adjusted *post hoc* mean-separation testing to determine the significance of every possible pair-wise comparison.

## 3 Results

### 3.1 Metabolic Imaging of Routine Culture SCC 74A and 74B Cells

To establish the typical variation in NAD(P)H intensity, lifetime, and bound NAD(P)H fraction, SCC74A and 74B cells were imaged under a variety of metabolic conditions including mitochondrial inhibition by rotenone and uncoupling by FCCP and analyzed with lifetime-fitting as well as phasor-based approaches. Although the bound NAD(P)H fraction and fluorescence lifetime represent the relative degree of enzyme binding, which changes with metabolic state, they are not dependent on the overall concentration of NAD(P)H. The fluorescence intensity can be used in conjunction with these measurements to assess the variation in NAD(P)H concentration since the intensity depends on the product of the concentration and the fluorescence lifetime.

**Fig. 3:**
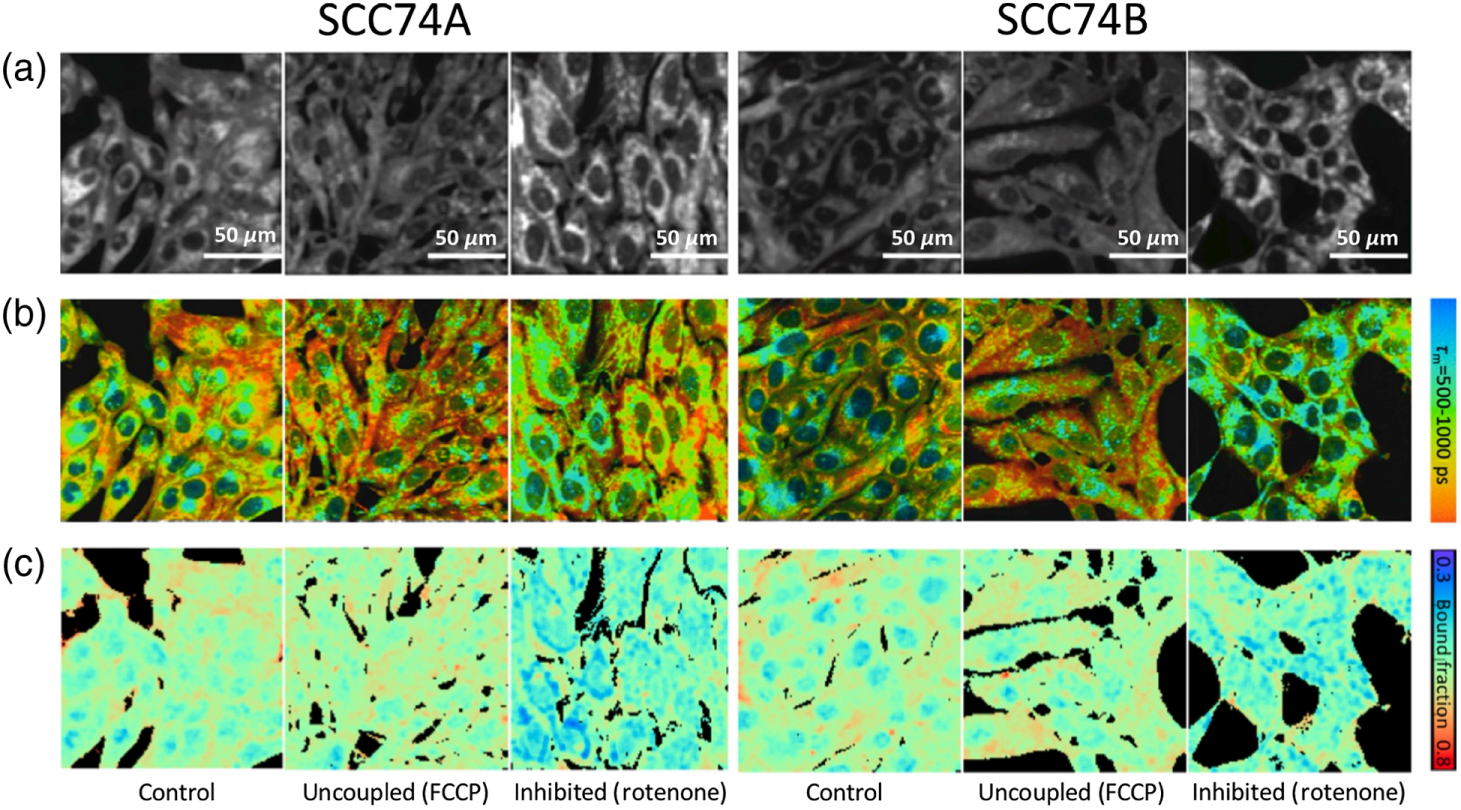
Effect of metabolic manipulation on routine cultured SCC74A and SCC74B. (a)–(c) Representative NAD(P)H intensity, lifetime, and bound NAD(P)H fraction images of untreated, uncoupled, and inhibited monolayer SCC 74A and SCC74B cells. Nuclei tend to have higher proportion of short-lifetime, free NAD(P)H; while longer lifetime, enzyme-bound NAD(P)H was observed in cytoplasmic compartment, representing OXPHOS activity in the mitochondria. Inhibition of ETC with rotenone resulted in increased free NAD(P)H, and thus lower NAD(P)H lifetime, whereas uncoupling had the opposite effect. As with lifetimes, rotenone treated cells had lower bound-NAD(P)H-fraction while FCCP treated cells had larger value. Scale bar is 50  μm.

Representative image panels are shown in Fig. 3 for both SCC74A and SCC74B. As expected, mitochondrial uncoupling using FCCP led to free NADH being rapidly consumed with higher electron transport chain activity, resulting in a decrease in intensity (top row) and a consistent increase in the mean NAD(P)H lifetime and bound NAD(P)H fraction (middle and bottom rows). In contrast, rotenone inhibition, specifically blocking NADH oxidation at complex I (NADH dehydrogenase), led to an increase in intensity (top row) and a decrease in both the average lifetime and bound NAD(P)H fraction (middle and bottom rows). Treatment with FCCP and rotenone revealed the metabolic dynamic range of two cell cultures, representing the maximum capacity of oxidative phosphorylation (NADH oxidation) and glycolysis/Krebs cycle (NAD+ reduction) of SCC74A and SCC74B, respectively.

**Fig. 4:**
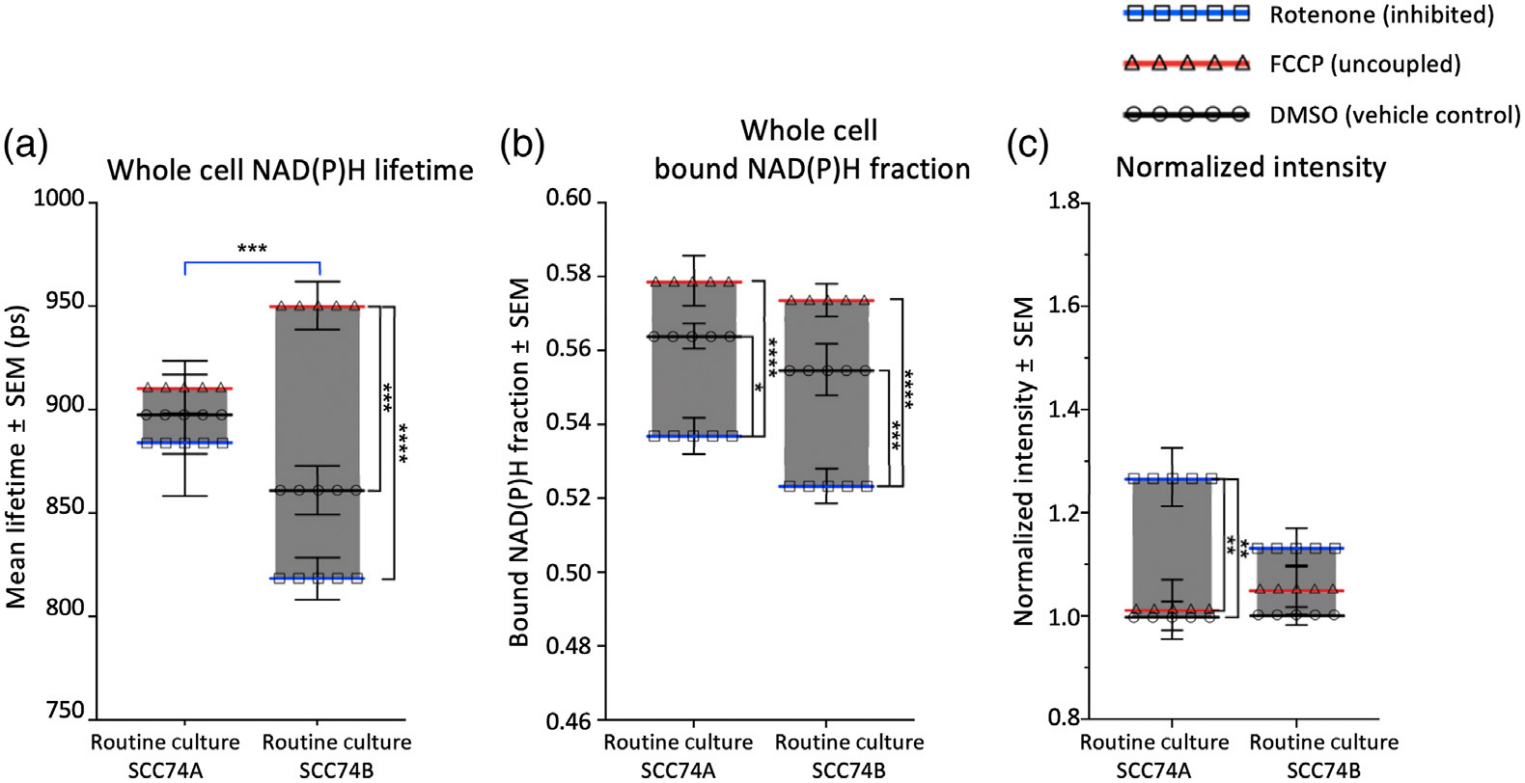
SCC74A and SCC74B showed different responses to metabolic treatments. (a), (b) In response to rotenone and FCCP treatment, SCC74B showed greater response in NAD(P)H binding activity, as shown through NAD(P)H mean lifetime and bound NAD(P)H fraction. (c) Meanwhile, SCC74A regulated NAD(P)H intensity, and hence, concentration. Error bars represent the SEM for 30 to 50 fields of view taken for each treatment from 6 (SCC74A) and 7 (SCC74B) replicates (*p<0.05; **p<0.01; ***p<0.001; ****p<0.0001 by factorial ANOVA and Tukey *post hoc* test).

Considering NAD(P)H binding revealed by both lifetime fitting and phasor FLIM analysis, SCC74B cells exhibited a greater range of metabolic variation compared to SCC74A. Between inhibited and uncoupled cells, the NAD(P)H lifetime varied by 125 ps in SCC74B cells, a 14% variation, compared to just 50 ps in SCC74A cells, a 3% variation [Fig. 4(a)]. Similarly, the bound NAD(P)H fraction varied more in SCC74B cells (6%) than in SCC74A cells (4%) between these two conditions [Fig. 4(b)]. However, the NAD(P)H intensity in SCC74B cells actually varied less than in SCC74A cells with metabolic treatments (a 13% variation for SCC74B and a 27% variation for SCC74A, compared to baseline intensity in respective control cells) [Fig. 4(c)].

Regarding the role of HER2 overexpression in cancer metabolism, SCC74B cells showed greater modulation in NAD(P)H binding activity while SCC74A cells showed greater modulation of NAD(P)H intensity in response to metabolic treatments of FCCP and rotenone. Both the NAD(P)H lifetime and bound fraction of routine cultured SCC74B cells significantly increased with FCCP treatment and decreased with rotenone treatment [Figs. 4(a) and 4(b)]. Meanwhile, both analysis approaches showed that NAD(P)H binding activity of SCC74A was less responsive to metabolic treatments, with the only statistically significant difference being the decrease in bound NAD(P)H fraction following rotenone [Figs. 4(a) and 4(b)]. Despite the changes in NAD(P)H lifetime and bound fraction with metabolic treatments, there was no significant difference in NAD(P)H intensity of treated SCC74B cells, suggesting that the impacts of FCCP and rotenone on NAD(P)H concentration were offset by their impacts on NAD(P)H binding and corresponding fluorescence quantum yield. Meanwhile, SCC74A cells had a significant increase in NAD(P)H intensity following rotenone treatment, implicating an increase in NAD(P)H concentration [Fig. 4(c)]. We observed no significant impact of the vehicle (DMSO) on NAD(P)H intensity or binding in either cell line (Fig. S1 in the Supplementary Material).

Taken together, in response to mitochondrial inhibition and uncoupling, SCC74A cells demonstrated a larger variation in NAD(P)H concentration, with relatively little change in the bound NAD(P)H fraction. In contrast, the high-HER2 SCC74B cells displayed a relatively stable NAD(P)H concentration but a larger variation in NAD(P)H binding activities upon metabolic treatments.

### 3.2 HER2 Inhibition Reduced NAD(P)H Lifetime and Bound NAD(P)H Fraction While Increasing NAD(P)H Intensity and Concentration in SCC74A and SCC74B Cells

**Fig. 5:**
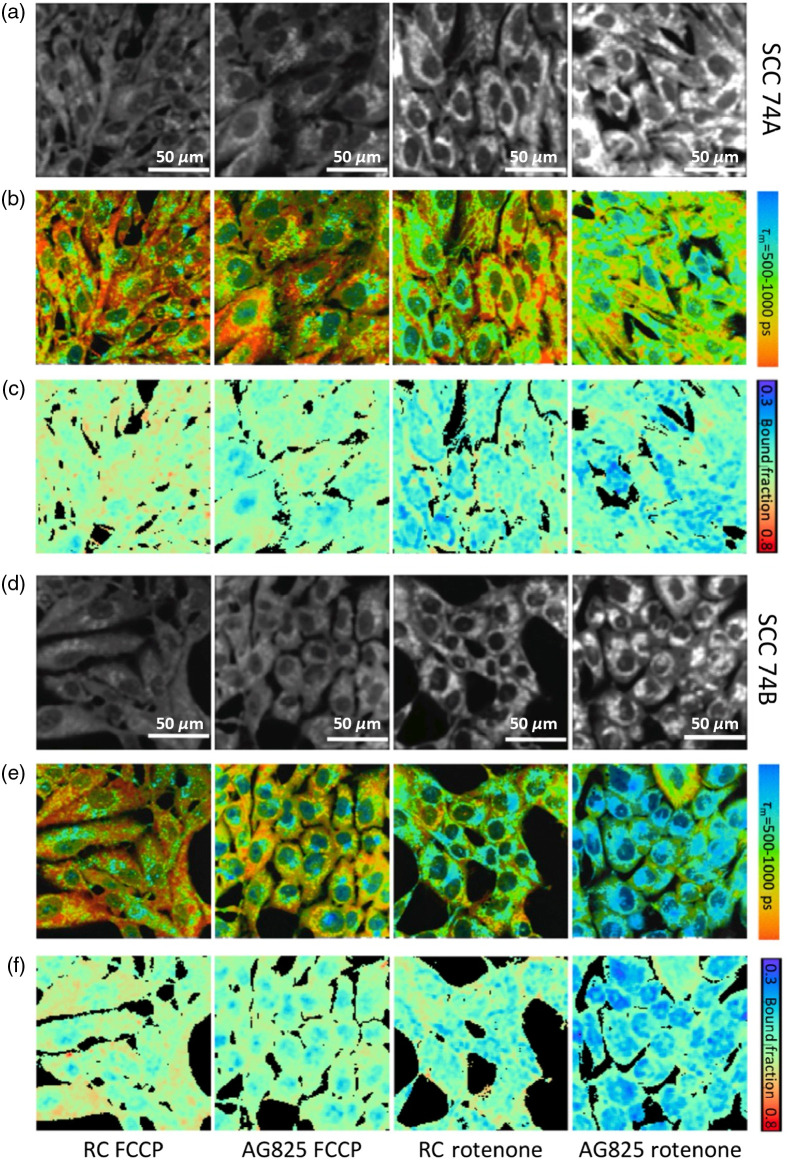
Effect of HER2 inhibitor AG825 on NAD(P)H intensity, lifetime, and bound fraction of (a)–(c) SCC 74A and (d)–(f) SCC 74B cells: (a), (d) intensity; (b), (e) lifetime; and (c), (f) bound fraction. Following HER2 inhibition by AG825, uncoupled and inhibited SCC74A and SCC74B (columns 2 and 4) showed decreased NAD(P)H lifetimes and bound fractions compared to RC (columns 1 and 3). Meanwhile, NAD(P)H intensity increased in both cell cultures following AG825 treatment, regardless of metabolic treatment. Scale bar is 50  μm.

**Fig. 6:**
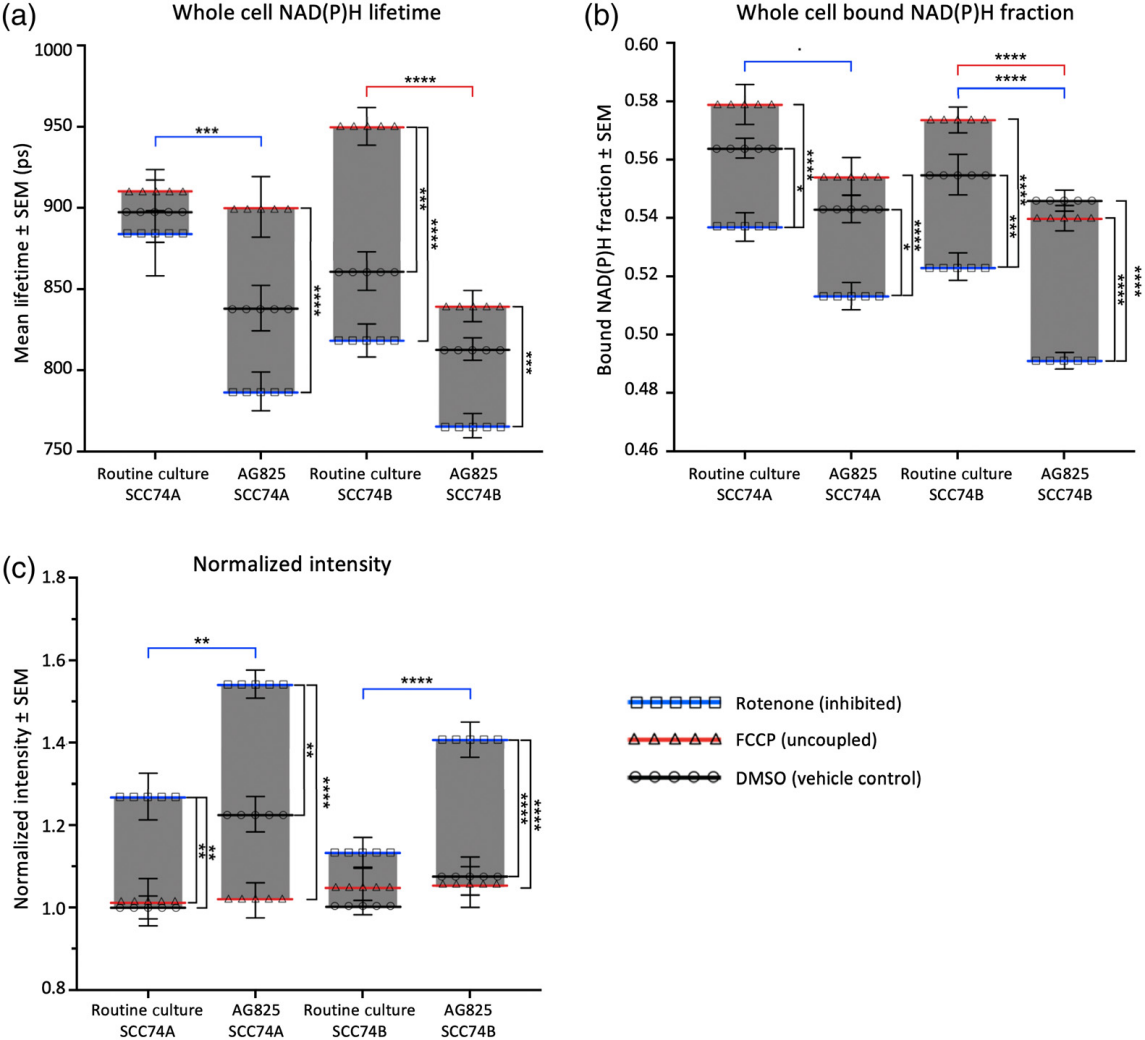
HER2 inhibition with AG825 reduced whole-cell NAD(P)H fluorescence lifetime while increasing NAD(P)H intensity in metabolically challenged SCC74A and SCC74B. Inhibited and uncoupled SCC74A and SCC74B showed significantly (a) lower lifetime and (b) bound NAD(P)H fraction following AG825 treatment compared to RC. AG825 did not have a significant impact on NAD(P)H lifetime of vehicle control SCC74A or SCC74B. (c) AG825 significantly increased NAD(P)H intensity in inhibited SCC74A and SCC74B. Error bars represented the SEM for 30 to 50 fields of view taken for each treatment from 6 (SCC74A) and 7 (SCC74B) replicates (*p<0.05; **p<0.01; ***p<0.001, ****p<0.0001 by factorial ANOVA and Tukey *post hoc* test).

To investigate the role of HER2 in regulating SCC metabolism, both SCC74A and SCC74B were treated with the HER2 inhibitor AG825, followed by either FCCP or rotenone. Whole cell analysis of NAD(P)H mean lifetime, bound NAD(P)H fraction and NAD(P)H intensity revealed changes in metabolism after 24 h of HER2 inhibition (Figs. 5 and 6). AG825 treatment significantly reduced the NAD(P)H lifetime and bound NAD(P)H fraction in both SCC74A and SCC74B cells [Figs. 5, 6(a), and 6(b)]. HER2-inhibited SCC74A and SCC74B cells also showed a remarkable reduction in NAD(P)H binding after rotenone and FCCP, compared to SCC cells with functional HER2.

In SCC74A, AG825 treatment sensitized cells to metabolic treatments, with FCCP treated cells having significantly higher NAD(P)H mean lifetime compared to rotenone-treated cells [Fig. 6(a)]. In both cell lines, following HER2 inhibition, FCCP treated cells did not show any significant differences in NAD(P)H mean lifetime, bound NAD(P)H fraction, or NAD(P)H intensity compared to vehicle control cells. NAD(P)H binding in HER2-inhibited SCC74B shifted further toward the maximally bound (FCCP-treated) states, as vehicle control cells had NAD(P)H lifetimes and bound fractions similar to uncoupled cells [Figs. 6(a) and 6(b)]. So while HER2 inhibition led to an overall reduction in the bound NAD(P)H fraction, attempts to increase the bound fraction by stimulating ETC oxidation of NAD(P)H with FCCP were not significant.

Although the overall variation of the bound NAD(P)H fraction due to metabolic perturbation did not change significantly in either cell line with HER2 inhibition, AG825 treatment significantly increased the NAD(P)H intensity of rotenone-inhibited cells in comparison to vehicle control and uncoupled cells [Fig. 6(c)]. However, HER2 inhibition had little effect on the NAD(P)H intensity of FCCP uncoupled cells. The decrease in the lifetime and increase in the intensity indicated an increased NAD(P)H concentration following HER2 inhibition, particularly when mitochondria were inhibited by rotenone.

### 3.3 Nuclear NAD(P)H Showed Greater Response to HER2 Inhibition, While Cytoplasmic NAD(P)H Was More Responsive to Metabolic Treatments

NAD(P)H lifetime and bound fraction images revealed considerably lower NAD(P)H intensity, lifetime, and bound fractions in the nuclear region compared to the cytoplasmic region (Fig. 3). Hence, significant treatment impacts might be masked by the inherent variation in NAD(P)H signal between the two cellular compartments. Furthermore, the roles of NAD(P)H in these two compartments are also drastically different. As our imaging method allows for subcellular resolution, we segmented and analyzed the nuclear and cytoplasmic regions of each cell separately to further investigate the effect of HER2 on these two cell lines.

**Fig. 7:**
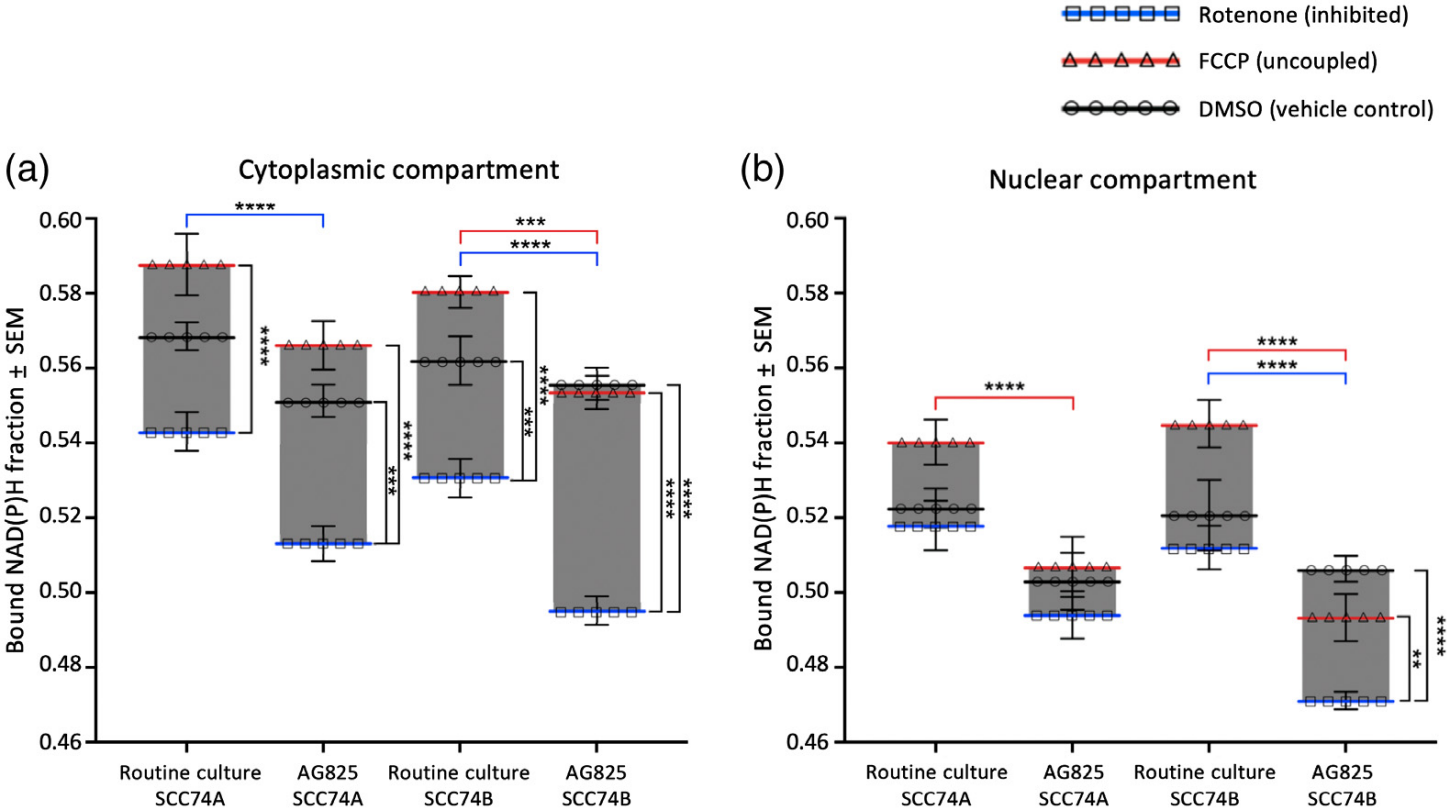
AG825 treatment reduced bound NAD(P)H fraction in both (a) cytoplasmic and (b) nuclear regions of metabolically challenged SCC74A and SCC74B cell lines. HER2 inhibition had an overall greater impact on SCC74B cell line compared to SCC74A. Nuclear compartments of both cell lines showed lower bound NAD(P)H fraction compared to cytoplasm. Nuclear NAD(P)H was more responsive to AG825 treatment than to metabolic treatments of FCCP and rotenone. Error bars represented the SEM for 30 to 50 fields of view taken for each treatment from 6 (SCC74A) and 7 (SCC74B) replicates (*p<0.05; **p<0.01; ***p<0.001; ****p<0.0001 by factorial ANOVA and Tukey *post hoc* test).

Both nuclear and cytoplasmic compartments of the two SCC cell lines showed a similar decrease in the bound NAD(P)H fraction with HER2 inhibition by AG825 (Fig. 7). This decrease was not significant in vehicle control cells. However, when challenged with either uncoupling by FCCP or inhibition of electron transport by rotenone, both compartments of HER2-inhibited SCC74B cells displayed a significantly lower bound NAD(P)H fraction compared to RC cells. In SCC74A, HER2 inhibition significantly decreased the bound NAD(P)H fraction in the cytoplasmic region (after metabolic inhibition) and the nuclear region (after uncoupling). For both cell lines, the range of the bound fraction established by metabolic treatments in the cytoplasmic compartment slightly increased following HER2 inhibition by AG825, mostly due to increased sensitivity to rotenone [Fig. 7(a)]. Uncoupling with FCCP did not have any significant impact on NAD(P)H binding in the cytoplasmic regions of either cell lines.

Generally, the range of bound NAD(P)H fraction, as established by metabolic treatments, was smaller in nuclear regions compared to cytoplasmic regions. The nuclear compartment of AG825-treated SCC74B showed increased sensitivity toward rotenone treatment, with significantly lower bound NAD(P)H fraction compared to vehicle control and uncoupled (FCCP-treated) cells. Besides that, no significant changes in NAD(P)H binding activities were observed following metabolic treatments in the nuclear regions of both cell lines under RC condition and of AG825-treated SCC74A [Fig. 7(b)]. Meanwhile, the percent change in the bound NAD(P)H fraction with AG825 treatment was similar, if not more substantial in the nuclear compartment compared to the cytoplasm for both cell lines (Table S1 in the Supplementary Material).

**Fig. 8:**
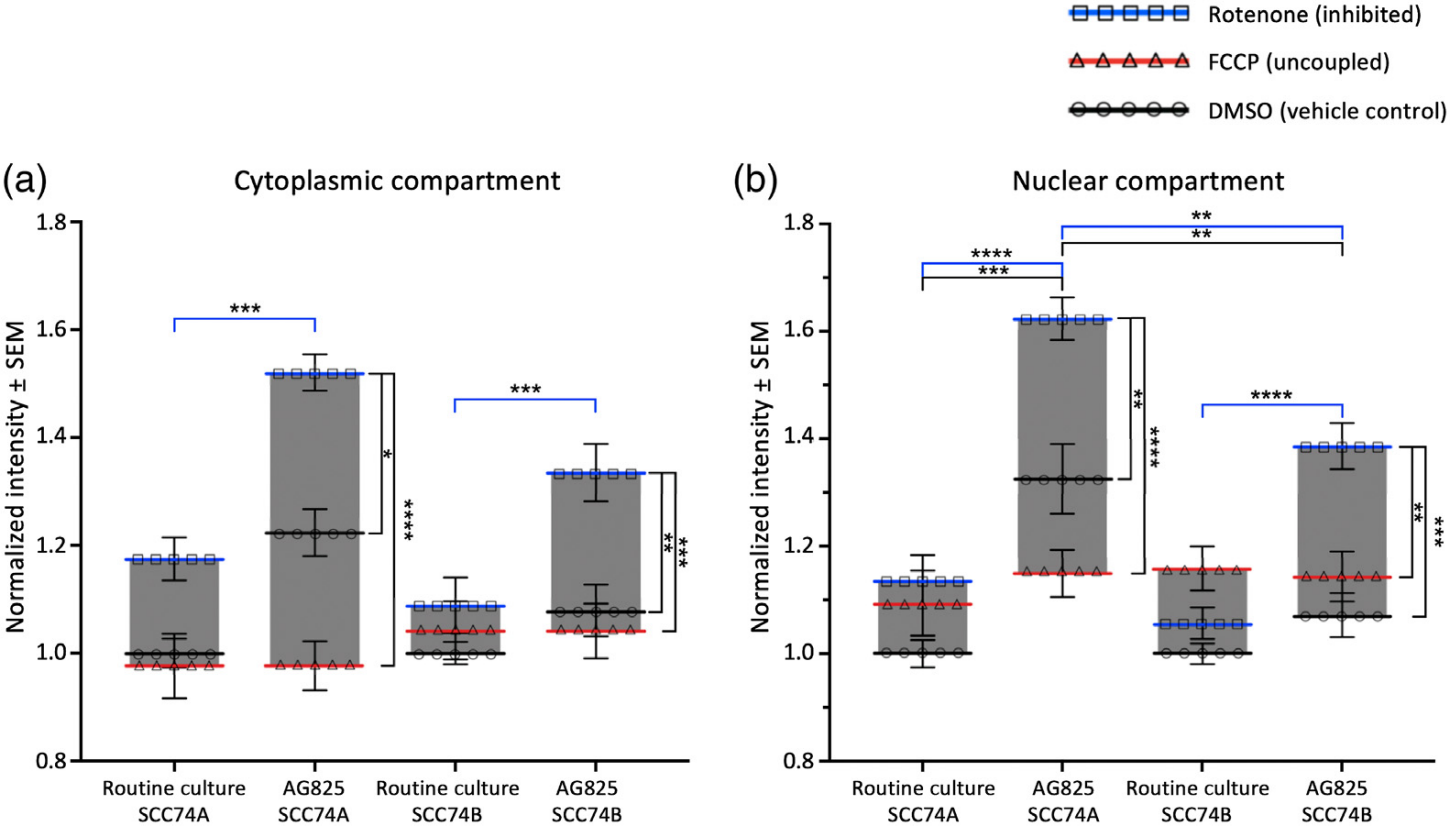
AG825 treatment significantly increased NAD(P)H intensity in both (a) cytoplasmic and (b) nuclear regions in SCC74A and SCC74B cell lines. Calibrated NAD(P)H intensity was normalized to the average intensity of vehicle control cells for each replicate. Significant increase in NAD(P)H intensity following AG825 treatment was observed in both compartments of metabolically inhibited SCC74A and SCC74B cells. Error bars are the SEM for 6 (SCC74A) or 7 (SCC74B) experiments with 5 to 7 images taken per treatment in each experiment (*p<0.05; **p<0.01; ***p<0.001; ****p<0.0001).

We also observed consistent increases in NAD(P)H intensity in both compartments of two cell lines following HER2-inhibition (Fig. 8). The increase was more significant in metabolically inhibited cells than in vehicle control cells and more remarkable in SCC74A than in SCC74B. In contrast, AG825 treatment did not significantly affect the NAD(P)H intensity of either cell line when uncoupled, in either compartment. Therefore, the difference in NAD(P)H intensity between fully reduced (rotenone) and fully oxidized (FCCP) states, greatly increases with AG825 treatment in both compartments. Using vehicle control cells as the metabolic baseline, we observed a greater increase in NAD(P)H intensity following rotenone treatment in HER2-inhibited SCC74A compared to HER2-inhibited SCC74B cells, especially in the nuclear region. After HER2 inhibition, vehicle control SCC74A cells also had significantly greater NAD(P)H intensity than SCC74B cells. The increase in intensity coupled with the decrease in bound NAD(P)H fraction suggested a substantial increase in free NAD(P)H concentration following HER2 inhibition, especially in metabolically inhibited cells treated with rotenone.

Overall, even though both the cytoplasmic and nuclear regions of the two cell lines show a similar pattern of response to HER2 inhibition with an increased proportion of free NAD(P)H, AG825 had a greater impact on the bound NAD(P)H fraction of high-HER2-expressing SCC74B than on its low-HER2 counterpart. Meanwhile, nuclear NAD(P)H showed similar and somewhat higher sensitivity to AG825 treatment but was less sensitive to metabolic perturbation compared to cytoplasmic NAD(P)H.

## 4 Discussion

### 4.1 Intensity-Based versus Fluorescence Lifetime-Based Metabolic Imaging

Non-invasive optical monitoring of metabolism based on the redox state of NADH and flavoproteins has been developed and implemented in a variety of ways since the time of its initial inception by Chance et al. more than 60 years ago.^11^ It has been well established in many systems that changes in NADH and flavoprotein fluorescence are inversely related, given that NAD(P)H is fluorescent in the reduced form while flavins are fluorescent when oxidized.^18^^,^^19^ By dividing the NAD(P)H fluorescence intensity by the oxidized flavin fluorescence intensity, the optical redox ratio can be calculated and used as a measure of cellular metabolism. Recent implementations of this technique have included sequential single-photon excitation at two separate excitation wavelengths with two-channel confocal detection, as well as multiphoton excitation, which has the advantage of using near-infrared light to better penetrate tissue *in vivo* with improved non-descanned detection sensitivity.^16^^,^^17^^,^^19^^,^^36^

While optical redox ratio measurements of two coupled fluorescence signals have been successful and have the advantage of improved sensitivity compared to monitoring either NAD(P)H or flavin alone, this comes with several potential disadvantages. To begin with, the cost of requiring either multiplexing two separate laser systems for simultaneous measurements, or wavelength tuning between sequential measurements to avoid bleed-through between the NAD(P)H and flavin fluorescence channels adds expense and is time-consuming. Furthermore, the flavin signal is often relatively weak in tissue compared to the NAD(P)H signal and this can contribute to noise in the redox ratio measurement. Also the relative intensity of these fluorophores also varies with imaging depth due to differential tissue penetration of both the excitation and emission light, making intensity-based measurements difficult to calibrate. More fundamentally, changes in fluorescence intensity can be difficult to interpret when changes in fluorophore binding lead to a corresponding change in the fluorescence quantum yield. For example, rotenone, by inhibiting the electron transport chain, increases NADH concentration while decreasing the average NADH fluorescence lifetime due to an increase in abundance of free NADH. Since the fluorescence intensity is proportional to the product of the lifetime and concentration, when these quantities change in opposite ways the intensity could either increase, decrease, or remain unchanged. Meanwhile, fluorescence lifetime is not masked by any additional variables and hence can serve as an independent indicator for cellular metabolism.

For these reasons, the strategy for metabolic imaging chosen for this present work is based on the combination of the NAD(P)H fluorescence lifetime and intensity. Fluorescence lifetime imaging offers the advantage of being sensitive to changes in the level of NAD(P)H binding to coenzymes, which changes with the balance between NAD+ reduction (via glycolysis and the Krebs cycle) and NADH oxidation (by the ETC). However, traditional lifetime-fitting approaches also suffer in at least two ways. First, they assume a model in order to extract a lifetime, and the quality of the model is dependent on the photon count of the fluorescence signal. Second, the process of lifetime fitting is iterative and time-consuming. Recently, phasor-based fluorescence imaging techniques have been gaining popularity in part because they circumvent these problems.^21^^,^^22^^,^^32^^,^^37^^–^^40^ Although these techniques do not explicitly determine a lifetime, the phasor coordinate is inherently dependent on the lifetime, and when calibrated against known standards, the shift in the phasor coordinates should be proportional to the fraction of the fluorescence signal associated with bound NAD(P)H, assuming an appropriate (biexponential) model.

The purpose of this study was to determine if it was possible to determine the influence of HER2 signaling in SCC, *in vitro*, with FLIM imaging and the phasor-based analysis approach. HER2 signaling is enhanced by exposure to UV light via ROS and is well known to stimulate abnormal levels of cellular proliferation leading to tumorigenesis. We wanted to determine how HER2 affected the capacity of cells to generate and utilize NADH by modifying the ETC activity through inhibition (rotenone) and uncoupling (FCCP). We also wanted to separately analyze changes in NADH fluorescence in nuclear and cytoplasmic regions, given the different roles that NADH plays in these regions. Finally, we combined and compared phasor-FLIM and traditional lifetime fitting analyses to verify that the two techniques led to a consistent interpretation of the data.

### 4.2 Comparison of NAD(P)H Lifetime Fitting versus Phasor FLIM Analysis

The two analyses described in Fig. 6 are qualitatively similar, though there are quantitative differences as well. As expected, AG825 inhibition of HER2 led to a reduction in both the mean lifetime and bound NAD(P)H fraction. However, no significant differences in the lifetime for RC SCC74A cells were observed following application of metabolic inhibitors and uncouplers, whereas the bound NAD(P)H fraction were significantly different. Furthermore, although only the lifetimes of the uncoupled SCC74B cells were significantly different with HER2 inhibition, the bound fractions for the rotenone-inhibited cells also differed significantly. Generally, the fractional variation (SEM/mean) in the bound fractions was on the order of 1% to 2%, whereas variation in the lifetimes was approximately of 3% to 6%. This likely reflects the greater sensitivity of the multiexponential fitting process to the number of detected photons.

### 4.3 Cytoplasmic and Nuclear NAD(P)H Signals May Reveal Different Roles in Cancer Cells

In both SCC74A and SCC74B cells, we observed and quantified NAD(P)H in both cytoplasmic and nuclear compartments of the cells. Although the majority of metabolic imaging studies have focused on either whole cell or cytoplasmic/mitochondrial NAD(P)H, by segmenting and analyzing both compartments, we were able to observe significant changes in nuclear NAD(P)H signal in response to perturbation. Although the direction of change is similar in the two compartments, cytoplasmic and nuclear NAD(P)H signals still display some differences in the magnitude of response to different types of perturbation.

In RC SCC74A and SCC74B cells, metabolic perturbation with either FCCP or rotenone significantly impacted the cytoplasmic bound NAD(P)H fraction, suggesting its primary involvement in metabolism. NAD(P)H binding did not change as significantly within the nucleus in response to these metabolic treatments. However, following HER2 inhibition, both cell lines show decreased NAD(P)H bound fraction in nuclear regions of metabolically challenged cells. The preferential response of nuclear NAD(P)H to HER2 inhibition compared to metabolic treatment suggests that nuclear NAD(P)H had a non-metabolic role downstream of HER2 signaling. It is also worth noticing that NAD(P)H intensity was not significantly affected by rotenone or FCCP in SCC74A and SCC74B cells with functional HER2. It is only after HER2 inhibition with AG825 that we observed significant changes in NAD(P)H intensity. Moreover, in all cases, nuclear NAD(P)H had a lower bound fraction and lower fluorescence intensity, indicating that the majority of nuclear NAD(P)H population is free. Previous studies have demonstrated that free nuclear NAD(P)H can function as a mediator of several transcriptional regulators, one of which is C terminal binding protein (Ctbp).^23^ As Ctbp plays an important role in promoting cell survival in carcinogenesis and its activation is highly dependent on nuclear free NAD(P)H, future study is warranted to investigate the relationship between nuclear NAD(P)H and Ctbp activity following HER2 inhibition in these two SCC cultures. Overall, our study suggests that nuclear NAD(P)H changes dynamically with HER2 inhibition, and hence, should be considered equally and separately from the cytoplasmic/mitochondrial signal.

### 4.4 HER2 Inhibition Appeared to Promote Increased Reliance on Oxidative Phosphorylation

In RC SCC74A and SCC74B cells, both compartments only displayed statistically significant differences in the bound NAD(P)H fraction and fluorescence intensity between inhibited and uncoupled states, the two opposite endpoints of the metabolic spectrum. The metabolic baseline of vehicle control cells lied within this range but was not significantly different from either endpoint, except for the case of the bound NAD(P)H fraction in the SCC74B cytoplasmic compartment. Following AG825 treatment, we notice that NAD(P)H signals in both compartments were more responsive to metabolic perturbation by rotenone. HER2-inhibited SCC74A and SCC74B cells had a significantly lower cytoplasmic bound NAD(P)H fraction and greater intensity following rotenone treatment, and SCC74B cells had significantly lower nuclear NAD(P)H intensity compared to SCC74A cells. Both cell lines rest closer to their maximal OXPHOS capacity following HER2 inhibition, and further away from their maximally reduced (glycolytic) state, though the shift was clearer in SCC74B than in SCC74A cells. In fact, inhibiting HER2 seems to lead to greater reliance on the ETC for ATP production, given that rotenone-inhibition of the ETC leads to a much greater NADH concentration in HER2 inhibited cells.

## 5 Conclusion

Skin cancer continues to be the most widespread form of cancer, with 1 in 5 people in the USA estimated to be diagnosed by the age of 70.^41^ Although the average 5-year survival rate is lower for melanoma, more than twice as many deaths are attributed to SCC due to its high incidence.^3^^,^^42^^,^^43^ The majority of these cancers are due to UV exposure, which is known to activate HER2. This in turn suppresses cell cycle arrest and promotes tumorigenesis. Although HER2 targeted therapies for cSCC have yet to be fully investigated, targeted therapies that specifically block HER2 have effectively reduced HNSCC tumorigenesis in animal models.^20^ Therefore, we conducted this study to determine if non-invasive measurements by NAD(P)H FLIM could be used to detect HER2-linked changes in metabolism both within the nucleus and the cytoplasm of HNSCC cells.

A variety of non-invasive metabolic imaging techniques based on the redox state of endogenous NADH and flavoproteins have been developed and implemented since Chance et al. first described their utility. These can broadly be categorized as techniques that assess the redox state by fluorescence intensity, notably the redox ratio, or by variation in fluorescence lifetime, which is sensitive to enzymatic binding and thereby linked to cellular metabolism. Within the category of fluorescence lifetime techniques, there are also a number of analysis techniques that have been effectively employed. Here we explored the combined use of the mean fluorescence lifetime, obtained by fitting time-correlated single-photon counting fluorescence decay curves to a sum of exponentials, and phasor FLIM analysis of the bound NAD(P)H fraction. Both have the advantage of being independent of total fluorescence intensity and require only a single laser excitation wavelength, which are particularly attractive for use *in vivo*. Although it is easy to find examples in the literature of successful studies that have used each of these individual techniques, it is more difficult to find quantitative comparisons using combinations of these techniques. In particular, we wondered if results obtained by fluorescence lifetime determination and phasor bound fraction would consistently result in the same biophysical interpretation, if there might be any practical advantage of one approach over the other, or if the combined analysis might provide further insight into the underlying mechanism of metabolic and epigenetic changes associated with HER2 expression in SCC.

As we explored the data, it quickly became clear that the mean fluorescence lifetime, the phasor bound fraction, and the fluorescence intensity all provided complimentary information that improves the overall interpretation of the data. Both FLIM techniques reflected consistent changes in NAD(P)H metabolism associated with inhibition of growth factor signaling in SCC cell lines with similar sensitivity. Furthermore, the change in the relative fluorescence intensity induced by metabolic and HER2 inhibition, though more difficult to accurately quantify, was particularly useful when used in conjunction with the mean lifetime to infer changes in NAD(P)H concentration.

Using mitochondrial inhibitors and uncouplers in conjunction with HER2 inhibition, the impact of growth factor signaling on the activity of the ETC could be assessed. Image segmentation was also particularly valuable since it allowed the differential response of nuclear and mitochondrial/cytoplasmic NAD(P)H to be discerned. While these compartments are connected within the cell, nuclear NAD(P)H clearly showed greater response to HER2 inhibition while cytoplasmic NAD(P)H was more responsive to metabolic treatments. Given the multifaceted role of NAD(P)H within the cell, it is important to consider changes in these compartments separately.

We chose the SCC74A and SCC74B cell lines for this study because previous work demonstrated elevated HER2 and EGFR in the more-aggressive SCC74B cell line (derived from a 1-year recurrence) compared to SCC74A cells (derived from the primary tumor at the same site).^20^^,^^25^ The combined analysis of NAD(P)H lifetime, bound fraction, and intensity consistently revealed that the SCC74A cells had a relatively large increase in NAD(P)H concentration compared to SCC74B cells when oxidation by the ETC was inhibited by rotenone. This suggests that oxidative phosphorylation is more significant in the low-HER2 SCC74A cell. HER2 inhibition reduced NAD(P)H lifetime and bound NAD(P)H fraction while increasing the fluorescence intensity. The combined analysis revealed an elevated NAD(P)H concentration as well as a greater proportion of the NAD(P)H signal due to free NADH following HER2 inhibition in both cell lines, though HER2 inhibition had a more significant effect on the high-HER2 SCC74B cells, as expected. Overall, the study shows inhibition of HER2 leads to an increase in ETC activity and greater reliance on oxidative phosphorylation.

This appears to be different than what has been reported by Walsh et al.^16^ for redox ratios in a variety of ER±, HER2±, and triple negative breast cancer cell lines, though they also observed a decrease in NAD(P)H lifetime with HER2-inhibition by trastuzumab. Lakner et al.^37^ used an *in vitro* model of colorectal carcinoma found that addition of growth factors caused a detectable decrease in the lifetime of NAD(P)H, an increase in the relative proportion of the short lifetime component, and a shift of the phasor distribution toward free NAD(P)H, which was interpreted as a trend toward glycolysis, though the change did not meet criteria for statistical significance.

It is worth noting that our culture conditions for these two cell lines allow for ample oxygen concentration that might not be representative of the *in vivo* tumor microenvironment and can directly affect cell growth and metabolism. This is likely the case for most literature results using cultured cells, suggesting metabolic and epigenetic signatures of cancer cells might be better evaluated under a hypoxic environment.^44^ Experiments to characterize change in NAD(P)H metabolism under hypoxia or during the development of UV-induced SCC are currently underway.

## Code, Data, and Materials Availability

Associated code and data can be made available upon request.

## References

[1] Iqbal N. Iqbal N., “Human epidermal growth factor receptor 2 (HER2) in cancers: overexpression and therapeutic implications,” Mol. Biol. Int. 2014, 852748 (2014). 10.1155/2014/852748 25276427 PMC4170925

[2] Johnson D. E. et al., “Head and neck squamous cell carcinoma,” Nat. Rev. Dis. Primers 6(1), 92 (2020). 10.1038/s41572-020-00224-3 33243986 PMC7944998

[3] Guy G. P. et al., “Vital signs: melanoma incidence and mortality trends and projections—United States, 1982–2030,” Morb. Mortal. Wkly. Rep. 64(21), 591–596 (2015). PMC4584771 26042651

[r4] Rogers H. W. et al., “Incidence estimate of nonmelanoma skin cancer (keratinocyte carcinomas) in the US population, 2012,” JAMA Dermatol. 151(10), 1081–1086 (2015). 10.1001/jamadermatol.2015.1187 25928283

[5] American Cancer Society, “Cancer facts & figures 2021,” https://www.cancer.org/research/cancer-facts-statistics/all-cancer-facts-figures/cancer-facts-figures-2019.html (2019). 10.6004/jadpro.2020.11.2.1 PMC7848816 33532112

[6] Riese D. J. Stern D. F., “Specificity within the EGF family/ErbB receptor family signaling network,” BioEssays 20(1), 41–48 (1998). BIOEEJ 0265-9247 10.1002/(SICI)1521-1878(199801)20:1<41::AID-BIES7>3.0.CO;2-V 9504046

[r7] Madson J. G. et al., “Erbb2 regulates inflammation and proliferation in the skin after ultraviolet irradiation,” Am. J. Pathol. 169(4), 1402–1414 (2006). AJPAA4 0002-9440 10.2353/ajpath.2006.060082 17003495 PMC1780187

[r8] Madson J. G. Hansen L. A., “Multiple mechanisms of Erbb2 action after ultraviolet irradiation of the skin,” Mol. Carcinog. 46(8), 624–628 (2007). MOCAE8 1098-2744 10.1002/mc.20335 17477367

[9] Rao V. H. et al., “Erbb2 up-regulation of ADAM12 expression accelerates skin cancer progression,” Mol. Carcinog. 56(5), 1162–1174 (2015). MOCAE8 1098-2744 10.1002/mc.22171 24798404

[10] Cantor J. R. Sabatini D. M., “Cancer cell metabolism: one hallmark, many faces,” Cancer Discov. 2(10), 881–898 (2012). 10.1158/2159-8290.CD-12-0345 23009760 PMC3491070

[11] Chance B. et al., “Intracellular oxidation-reduction states in vivo,” Science 137(3529), 499–508 (1962). SCIEAS 0036-8075 10.1126/science.137.3529.499 13878016

[12] Chance B. et al., “Oxidation-reduction ratio studies of mitochondria in freeze-trapped samples. NADH and flavoprotein fluorescence signals,” J. Biol. Chem. 254(11), 4764–4771 (1979). 220260

[13] Zhang Z. et al., “Metabolic imaging of tumors using intrinsic and extrinsic fluorescent markers,” Biosens. Bioelectron. 20(3), 643–650 (2004). BBIOE4 0956-5663 10.1016/j.bios.2004.03.034 15494250

[r14] Ramanujam N. et al., “Low temperature fluorescence imaging of freeze-trapped human cervical tissues,” Opt. Express 8(6), 335–343 (2001). OPEXFF 1094-4087 10.1364/OE.8.000335 19417824

[15] Alhallak K. et al., “Optical redox ratio identifies metastatic potential-dependent changes in breast cancer cell metabolism,” Biomed. Opt. Express 7(11), 4364–4374 (2016). BOEICL 2156-7085 10.1364/BOE.7.004364 27895979 PMC5119579

[16] Walsh A. et al., “Optical imaging of metabolism in HER2 overexpressing breast cancer cells,” Biomed. Opt. Express 3(1), 75–85 (2012). BOEICL 2156-7085 10.1364/BOE.3.000075 22254170 PMC3255344

[17] Walsh A. J. et al., “Optical metabolic imaging identifies glycolytic levels, subtypes, and early-treatment response in breast cancer,” Cancer Res. 73(20), 6164–6174 (2013). CNREA8 0008-5472 10.1158/0008-5472.CAN-13-0527 24130112 PMC3801432

[18] Nichols M. G. et al., “Autofluorescence lifetime imaging,” in Natural Biomarkers for Cellular Metabolism, Ghukasyan V. Heikal A., Eds., pp. 104–133, CRC Press, Boca Raton, Florida (2014).

[19] Tiede L. M. et al., “Determination of hair cell metabolic state in isolated cochlear preparations by two-photon microscopy,” J. Biomed. Opt. 12(2), 021004 (2007). JBOPFO 1083-3668 10.1117/1.2714777 17477711 PMC1992521

[20] Rao V. H. et al., “A positive feedback loop between HER2 and ADAM12 in human head and neck cancer cells increases migration and invasion,” Oncogene 31(23), 2888–2898 (2012). ONCNES 0950-9232 10.1038/onc.2011.460 21986939 PMC3302945

[21] Digman M. A. et al., “The phasor approach to fluorescence lifetime imaging analysis,” Biophys. J. 94(2), L14–L16 (2008). BIOJAU 0006-3495 10.1529/biophysj.107.120154 17981902 PMC2157251

[22] Stringari C. et al., “Phasor approach to fluorescence lifetime microscopy distinguishes different metabolic states of germ cells in a live tissue,” Proc. Natl. Acad. Sci. U. S. A. 108(33), 13582–13587 (2011). 10.1073/pnas.1108161108 21808026 PMC3158156

[23] Zhang Q. Piston D. W. Goodman R. H., “Regulation of corepressor function by nuclear NADH,” Science 295(5561), 1895–1897 (2002). SCIEAS 0036-8075 10.1126/science.1069300 11847309

[24] Fjeld C. C. Birdsong W. T. Goodman R. H., “Differential binding of NAD+ and NADH allows the transcriptional corepressor carboxyl-terminal binding protein to serve as a metabolic sensor,” Proc. Natl. Acad. Sci. U. S. A. 100(16), 9202–9207 (2003). 10.1073/pnas.1633591100 12872005 PMC170896

[25] Worsham M. J. et al., “Chromosomal aberrations identified in culture of squamous carcinomas are confirmed by fluorescence in situ hybridisation,” Mol. Pathol. 52(1), 42–46 (1999). 10.1136/mp.52.1.42 10439839 PMC395670

[26] Zholudeva L. V. et al., “Gentamicin differentially alters cellular metabolism of cochlear hair cells as revealed by NAD(P)H fluorescence lifetime imaging,” J. Biomed. Opt. 20(5), 051032 (2015). JBOPFO 1083-3668 10.1117/1.JBO.20.5.051032 25688541 PMC4405084

[27] Vergen J. et al., “Metabolic imaging using two-photon excited NADH intensity and fluorescence lifetime imaging,” Microsc. Microanal. 18(4), 761–770 (2012). MIMIF7 1431-9276 10.1017/S1431927612000529 22832200 PMC3842212

[28] Štefl M. et al., “Applications of phasors to in vitro time-resolved fluorescence measurements,” Anal. Biochem. 410(1), 62–69 (2011). ANBCA2 0003-2697 10.1016/j.ab.2010.11.010 21078290 PMC3065364

[29] Chen Y. C. et al., “General concerns of FLIM data representation and analysis: frequency-domain model-free analysis,” in FLIM Microscopy in Biology and Medicine, Periasamy A. Clegg R. M., Eds., pp. 291–339, Chapman & Hall/CRC, New York (2010)

[r30] Vallmitjana A. et al., “Resolution of 4 components in the same pixel in FLIM images using the phasor approach,” Methods Appl. Fluoresc. 8(3), 035001 (2020). 10.1088/2050-6120/ab8570 32235070

[r31] Ranjit S. et al., “Determination of the metabolic index using the fluorescence lifetime of free and bound nicotinamide adenine dinucleotide using the phasor approach,” J. Biophotonics 12(11), e201900156 (2019). 10.1002/jbio.201900156 31194290 PMC6842045

[32] Malacrida L. et al., “The phasor plot: a universal circle to advance fluorescence lifetime analysis and interpretation,” Annu. Rev. Biophys. 50(1), 575–593 (2021). ARBNCV 1936-122X 10.1146/annurev-biophys-062920-063631 33957055

[33] Datta R. et al., “Fluorescence lifetime imaging of endogenous biomarker of oxidative stress,” Sci. Rep. 5(1), 9848 (2015). SRCEC3 2045-2322 10.1038/srep09848 25993434 PMC4438616

[34] Aguilar-Arnal L. et al., “Spatial dynamics of SIRT1 and the subnuclear distribution of NADH species,” Proc. Natl. Acad. Sci. U. S. A. 113(45), 12715–12720 (2016). 10.1073/pnas.1609227113 27791113 PMC5111721

[35] R Core Team, R: A Language and Environment for Statistical Computing, R Foundation for Statistical Computing (2018).

[36] Huang S. Heikal A. A. Webb W. W., “Two-photon fluorescence spectroscopy and microscopy of NAD(P)H and flavoprotein,” 82(5), 2811–2825 (2002). 10.1016/S0006-3495(02)75621-X PMC1302068 11964266

[37] Lakner P. H. et al., “Applying phasor approach analysis of multiphoton FLIM measurements to probe the metabolic activity of three-dimensional in vitro cell culture models,” Sci. Rep. 7(1), 1–11 (2017). SRCEC3 2045-2322 10.1038/srep42730 28211922 PMC5304149

[r38] Datta R. et al., “Fluorescence lifetime imaging microscopy: fundamentals and advances in instrumentation, analysis, and applications,” J. Biomed. Opt. 25(7), 071203 (2020). JBOPFO 1083-3668 10.1117/1.JBO.25.7.071203 PMC7219965 32406215

[r39] Evers M. et al., “Enhanced quantification of metabolic activity for individual adipocytes by label-free FLIM,” Sci. Rep. 8, 8757 (2018). SRCEC3 2045-2322 10.1038/s41598-018-27093-x 29884881 PMC5993796

[40] Fereidouni F. et al., “Rapid fluorescence lifetime estimation with modified phasor approach and Laguerre deconvolution: a comparative study,” Methods Appl. Fluoresc. 5(3), 035003 (2017). 10.1088/2050-6120/aa7b62 28644150 PMC6043162

[41] “Cancer facts and figures 2021,” American Cancer Society, https://www.cancer.org/content/dam/cancer-org/research/cancer-facts-and-statistics/annual-cancer-facts-and-figures/2021/cancer-facts-and-figures-2021.pdf (accessed 19 August 2021).

[42] “Our new approach to a challenging skin cancer statistic,” The Skin Cancer Foundation, https://www.skincancer.org/blog/our-new-approach-to-a-challenging-skin-cancer-statistic/ (accessed 19 August 2021).

[43] Mansouri B. Housewright C., “The treatment of actinic keratoses—the rule rather than the exception,” J. Am. Acad. Dermatol. 153(11), 1200 (2017). JAADDB 0190-9622 10.1001/jamadermatol.2017.3395 28975200

[44] Chen Y. et al., “Oxygen consumption can regulate the growth of tumors, a new perspective on the Warburg effect,” PLoS One 4(9), e7033 (2009). POLNCL 1932-6203 10.1371/journal.pone.0007033 19753307 PMC2737639

