# Supplementary material for: Development and characterization of phasor-based analysis for FLIM to evaluate the metabolic and epigenetic impact of HER2 inhibition on squamous cell carcinoma cultures
Source: J Biomed Opt. 2021 Oct 9;26(10):106501. doi: 10.1117/1.JBO.26.10.106501 (PMC8501457; doi:10.1117/1.JBO.26.10.106501)
Supplement: Supplementary file 1 [file JBO_026_106501_SD001.docx]

Supplementary Material


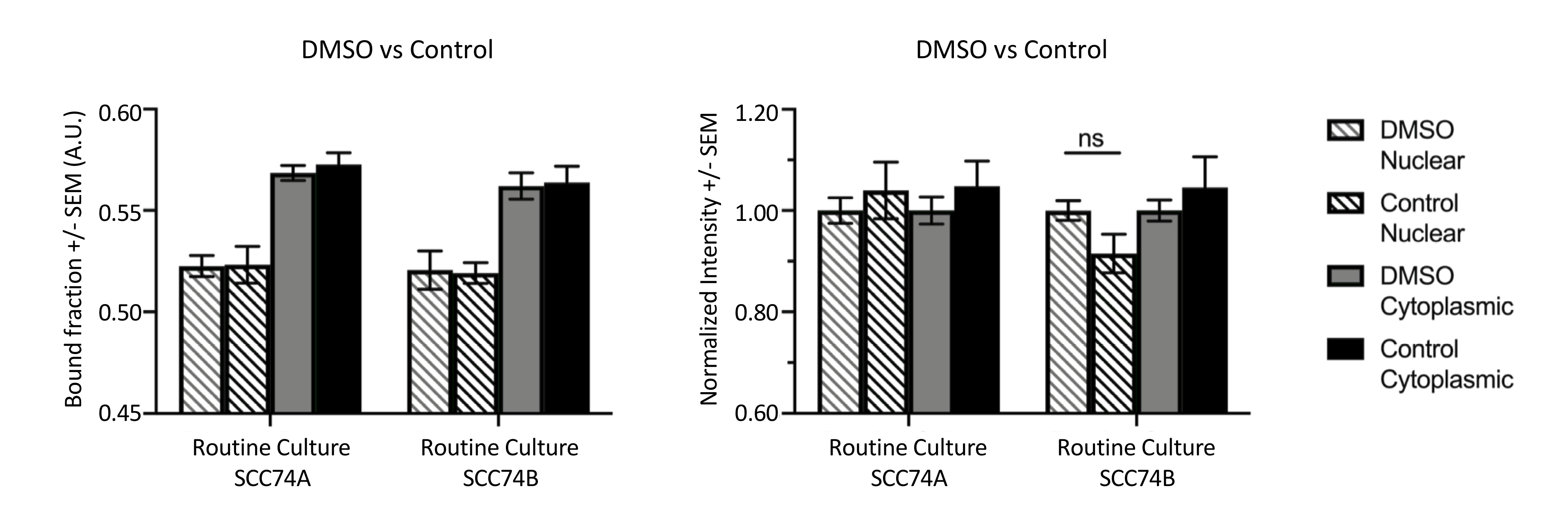


**Figure S1. DMSO did not have any significant effect on cellular metabolism in either compartment of two cell lines**. Bound NAD(P)H fraction and NAD(P)H intensity were the same for Control and DMSO treated cells.

| Metabolic Treatment | AG825 (+/-) | SCC 74A | | | | SCC 74B | | | |
| --- | --- | --- | --- | --- | --- | --- | --- | --- | --- |
|  |  | Cytoplasm | | Nucleus | | Cytoplasm | | Nucleus | |
|  |  | Bound fraction | SEM | Bound- fraction | SEM | Bound fraction | SEM | Bound fraction | SEM |
| DMSO | - | 0.569 | 0.004 | 0.523 | 0.005 | 0.562 | 0.006 | 0.521 | 0.009 |
|  | + | 0.551 | 0.004 | 0.503 | 0.008 | 0.556 | 0.004 | 0.506 | 0.003 |
|  | % change | -3.03 | | -3.76 | | -1.11 | | -2.75 | |
| 10μM FCCP | - | 0.588 | 0.008 | 0.540 | 0.006 | 0.580 | 0.004 | 0.545 | 0.006 |
|  | + | 0.566 | 0.006 | 0.507 | 0.008 | 0.553 | 0.004 | 0.493 | 0.006 |
|  | % change | -3.67 | | -6.17 | | -4.63 | | -9.51 | |
| 1μM rotenone | - | 0.543 | 0.005 | 0.518 | 0.007 | 0.531 | 0.005 | 0.512 | 0.006 |
|  | + | 0.513 | 0.005 | 0.494 | 0.006 | 0.495 | 0.004 | 0.471 | 0.002 |
|  | % change | -5.52 | | -4.62 | | -6.68 | | -7.99 | |

**Table S1. AG825 decreases bound NAD(P)H fraction in both cytoplasmic and nuclear regions of SCC74A and SCC74B**. The percentage change due to AG825 treatment is greater in SCC74B compared to SCC74A in metabolically disrupted (FCCP-treated and rotenone-treated) cells. Within the same cell lines and under the same metabolic treatment, percentage decrease in Bound NAD(P)H fraction is greater in the nuclear compared to cytoplasmic regions.
